# Think3d!: Improving mathematics learning through embodied spatial training

**DOI:** 10.1186/s41235-017-0052-9

**Published:** 2017-02-20

**Authors:** Heather Burte, Aaron L. Gardony, Allyson Hutton, Holly A. Taylor

**Affiliations:** 1 Department of Psychology, Tufts University, 490 Boston Ave, Medford, MA 02155 USA; 2 Center for Applied Brain & Cognitive Sciences, 200 Boston Ave, Medford, MA 02155 USA; 3 Cognitive Science Team, U.S. Army Natick Soldier Research, Development, and Engineering Center, Natick, MA 01760 USA; 4 Think3d!, 1018 Maple Lane, Davis, CA 95616 USA

**Keywords:** Mathematics, Embodied, Spatial thinking, Spatial visualization, Elementary students, Origami

## Abstract

Spatial thinking skills positively relate to Science, Technology, Engineering, and Math (STEM) outcomes, but spatial training is largely absent in elementary school. Elementary school is a time when children develop foundational cognitive skills that will support STEM learning throughout their education. Spatial thinking should be considered a foundational cognitive skill. The present research examined the impact of an embodied spatial training program on elementary students’ spatial and mathematical thinking. Students in rural elementary schools completed spatial and math assessments prior to and after participating in an origami and pop-up paper engineering-based program, called Think3d!. Think3d! uses embodied tasks, such as folding and cutting paper, to train two-dimensional to three-dimensional spatial thinking. Analyses explored spatial thinking gains, mathematics gains – specifically for problem types expected to show gains from spatial training – and factors predicting mathematics gains. Results showed spatial thinking gains in two assessments. Using a math categorization to target problems more and less likely to be impacted by spatial training, we found that all students improved on real-world math problems and older students improved on visual and spatial math problems. Further, the results are suggestive of developmental time points for implementing embodied spatial training related to applying spatial thinking to math. Finally, the spatial thinking assessment that was most highly related to training activities also predicted math performance gains. Future research should explore developmental issues related to how embodied spatial training might support STEM learning and outcomes.

## Significance

A Science, Technology, Engineering, and Math (STEM)-educated workforce is essential to support future growth in science and technology; however, not enough students are entering and/or being retained in STEM fields to support the demand (President’s Council of Advisors on Science and Technology, 2012). While education reforms in college may improve retention, education reforms before college would likely bolster interest in STEM fields and expand the pipeline of college students who are effective STEM learners. Spatial thinking training might foster interest in STEM fields before college, as spatial thinking skills – being able to mentally and/or physically manipulate the location and/or movement of people and/or objects (National Research Council, 2006) – is an important predictor of interest, education, and careers in STEM fields (Wai, Lubinski, & Benbow, 2009). In this work, we investigated spatial training using an embodied spatial training program called Think3d!. Think3d! combines embodied activities, such as folding and cutting paper and diagramming, with spatial training, such as interpreting and creating representations of two-dimensional (2D) and three-dimensional (3D) objects, to foster elementary students’ spatial and mathematical reasoning. After participating in Think3d!, all students showed gains in spatial visualization, real-world math problems, and for older grades, visual and spatial math problems, suggesting that Think3d! provides a targeted intervention for spatial and mathematical reasoning.

## Background

**Fig. 1:**
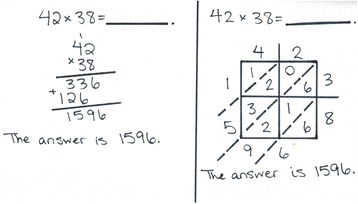
Solving a vertical multiplication problem by rewriting it horizontally (*left*) and using a lattice (*right*)

Consider how you would solve this problem: 42 × 38 = ___. Most people would solve it by re-writing it vertically (Fig. 1), then multiplying 42 by 8, 42 by 3, and finally adding their products together. An alternative approach is to use a lattice (Fig. 1), in which you draw a 2 × 2 lattice with the 42 on top and 38 on the side. Each digit is then multiplied to fill in the lattice, and the answer is obtained by adding the digits within the lattice along each diagonal. Both approaches are algorithmic, requiring a series of steps, but also spatial, as there is movement top to bottom, left to right, and, in the case of the lattice, diagonally. So, while elementary mathematics sometimes involves learning math facts (e.g. the multiplication table) and procedures for solving problems, mathematics can also be very spatial.

Spatial thinking involves using spatial relations between objects or spaces to reason and understand. Spatial thinking can be applied to problem-solving in many domains, including the mathematics problems described above. Individuals better at spatial thinking, as measured through cognitive tasks such as Mental Paper Folding (Shepard & Feng, 1972), Mental Rotation (Shepard & Metzler, 1971), and Purdue Visualizations of Rotations (Guay, 1976; henceforth referred to as Purdue Rotations), are arguably considered to have better spatial skills.

Spatial skills seem to play a unique role in learning and developing expertise in STEM disciplines (Wai et al., 2009). In their review chapter, Uttal and Cohen (2012) propose that spatial training may give students the spatial skills needed to conceptualize STEM ideas and make progress in their field. Some support for the connection between spatial skills and STEM comes from longitudinal studies. The spatial skills of intellectually talented high school students predicted their interest in and pursuit of a STEM career, more so than their verbal and quantitative abilities (Shea, Lubinski, & Benbow, 2001). Thirty years later, those students who had better spatial and math skills reported engineering, computer science, and/or mathematics as their favorite courses, college majors, and career pursuits, compared to individuals with better verbal abilities (Lubinski & Benbow, 2006). Further follow-up revealed a link between better spatial skills and career success as measured by patents and peer-reviewed publications, especially in STEM fields (Kell, Lubinski, Benbow, & Steiger, 2013). The spatial-STEM connection is not limited to talented students. Using a stratified random sample of high school students, spatial skills again predicted STEM education and career choices (Wai et al., 2009). This research suggests a provocative connection between spatial skills in high school and STEM outcomes. However, high school may be too late for developing the spatial skills needed for STEM education and career choices. Developing spatial skills well before high school may have a more pronounced impact on STEM outcomes. Despite the seemingly obviousness of this idea, spatial training is largely absent from elementary classrooms (National Research Council, 2006). The present work asked whether spatial training can be used to improve STEM understanding, particularly in mathematics, before college.

Researchers have started to explore how spatial skills impact STEM reasoning, with findings suggesting that some areas of mathematics can benefit from spatial thinking. One study found that children’s spatial skills in grades 1 and 2 predicted improvements in linear number line understanding over a year and this improvement mediated calculation skills three years later (Gunderson, Ramirez, Beilock, & Levine, 2012).

While the previously cited literature suggests a strong connection between spatial skills and STEM outcomes, some recent work has focused on how spatial training might impact both spatial thinking and STEM outcomes. For spatial thinking, a meta-analysis of spatial training studies found stable and consistent positive training effects for both trained and untrained spatial tasks (Uttal et al., 2013; Uttal, Miller, & Newcombe, 2013). Training effects lasted even after a relatively substantial delay.

The impact of spatial training on STEM outcomes is less clear. In their review, Stieff and Uttal (2015) suggested that spatial training shows promise for improving STEM success. However, they also noted that few studies have directly addressed this question. Of those that have, methodological issues often precluded conclusions, suggesting the need for more research. In a recent study that followed gifted STEM undergraduates, Miller and Halpern (2013) found potentially promising, but mixed, results. They found that spatial training improved mental rotation and spatial visualization skills and reduced gender differences on these spatial tasks. They also showed improved physics learning outcomes, but only for a relatively narrow range of problems and only short-term improvements.

Only one study has examined how spatial training impacts STEM reasoning in younger children. Cheng and Mix (2014) trained elementary students using either mental rotation or crossword puzzles (control). Changes between pre-test and post-test math (two-digit and three-digit calculation and missing term problems) and spatial (mental rotation and spatial relations) assessments showed improvements for both, but math improvements were limited to missing term problems. The authors suggested that mental rotation training allowed students to spatially re-arrange missing term equations into standard equation format (e.g. 7 + __ = 9 into 9 − 7 = __).

In summary, results from the studies explicitly exploring how spatial training impacts STEM thinking have shown variable outcomes. Most showed improvement on some, but not all, STEM problem types. This variability may arise from how STEM outcomes have been assessed. One would not expect spatial thinking to be used in all problems across all STEM fields. Consistent with this point, studies rarely showed overall course grade improvements, probably because many factors, one potentially being spatial skills, likely impacted STEM course grades. Identifying concepts and problem types that more likely to involve spatial thinking within a STEM discipline may provide a more targeted approach to understanding how spatial skills impact STEM learning.

The nature of the spatial training may also affect its impact. Embodied spatial training, in contrast with mental rotation practice, may more strongly affect spatial thinking. Embodied or grounded cognition posits that cognition is “grounded” within the sensory modalities, bodily states, and actions and so engages perceptual and action simulations (Barsalou, 2008, 2010). To date, embodied activities have been explored for spatial and mathematical reasoning separately. In support of embodied training contributing to spatial thinking gains, sports involving physical rotation (e.g. wrestling), lead to gains in mental rotation compared to sports without this property (Moreau, Clerc, Mansy-Dannay, & Guerrien, 2012). Further, stimuli that promote mental simulation of body rotation, for example by adding body parts to block configurations, improves mental transformations, but only when the body transformations are physically possible (Amorim, Isableu, & Jarraya, 2006).

For embodied approaches to teaching mathematics, researchers argue that students would benefit from learning opportunities that “ground” mathematics knowledge in direct experience (Ionescu & Vasc, 2014) and externalize mathematics concepts using representations (Pape & Tchoshanov, 2001). Embodied activities, such as walking along a number line, have been shown to improve number skills (Link, Moeller, Huber, Fischer, & Nuerk, 2013). Similarly, children understand magnitude better after playing number games using full-body movements on a digital dance mat, compared to working on a tablet computer (Fischer, Moeller, Bientzle, Cress, & Nuerk, 2011). Board games requiring piece movement also positively affect math understanding. Specifically, playing linear board games improved preschoolers’ understanding of magnitude and estimation over playing card games (Whyte & Bull, 2008). Moeller et al. (2012) suggest that early embodied learning, specifically counting on fingers, then evokes a finger-based representation of numbers whenever they are encountered. Many embodied math activities have a clear spatial component, as well, further strengthening the importance of spatial thinking in conceptualizing math.

In summary, educational and cognitive accounts converge to suggest that embodied spatial training can potentially improve spatial thinking and, in turn, mathematical reasoning. Longitudinal studies confirm spatial skills’ unique role in developing STEM expertise in college and beyond. Training studies demonstrate the potential of spatial interventions to improve both spatial thinking and targeted STEM outcomes in elementary and older students. Further, embodied math activities, which frequently have spatial components, promote mathematical concept understanding. Finally, in one study combining spatial training and math understanding, Cheng and Mix (2014) showed spatial and specific math improvements after spatial training. This study provided preliminary evidence that brief spatial training (one session) leads to targeted math improvements (missing term problems).

Building upon this study and other previous work, the present work examined the impact of a longer training program on spatial thinking and targeted STEM, in this case math, assessments. Our targeted approach tested a math categorization designed to identify math problem types more likely to be impacted by spatial training (Burte, Gardony, Hutton, & Taylor: Elementary teachers’ attitudes and beliefs towards spatial thinking in mathematics, submitted). At the same time, this categorization used a more diverse set of math problems than seen in previous studies. For the present work, we hypothesized that embodied spatial training targeting elementary-aged students would improve spatial thinking and mathematical reasoning. What follows is an overview of such a program, Think3d!, and a mathematical categorization used to assess targeted improvements in mathematical reasoning after embodied spatial training.

### The Think3d! program and its previous implementation

Think3d! (http://www.think3d.us.com) is designed to develop and strengthen visuospatial thinking through origami and pop-up paper engineering activities (Taylor & Hutton, 2013). Physically folding/unfolding and cutting paper and interpreting and constructing diagrams provide embodied approaches to spatial thinking (Taylor & Tenbrink, 2013, Tenbrink & Taylor, 2015). Such physical actions have been shown to improve children’s spatial thinking (Chu & Kita, 2011; Ping, Ratliff, Hickey, & Levine, 2011). The program’s structure involves diagram interpretation, 2D to 3D comparisons, and problem-solving, all embedded within the task of constructing paper objects. This allows for transition from physically supported to internalized spatial thinking (Novack, Congdon, Hemani-Lopez, & Goldin-Meadow, 2014).

In addition to embodying spatial thinking, the program employs a structure designed to maximize cognitive impact. Successive lessons for origami and paper engineering first give students cognitive tools, primarily focused on interpreting 2D diagrams of 3D information, and then ask them to enact their newly found understanding through exploration and experimentation. Early lessons use labeled diagrams to introduce a visual language for spatial thinking and to provide diagram interpretation practice. Later lessons have students reverse engineer fold and/or fold-cut sequence to reproduce a completed paper object. Students then construct diagrams to explain how to make paper objects to their peers. Instruction is not prescriptive, but rather exploratory. Students work in small groups, providing hints and feedback to one another. Mistakes come only at the cost of reaching for a new piece of paper, allowing students to easily enact and test their spatial thinking hypotheses in iterative ways.

Think3d! focuses on elementary students as this is likely an ideal time to introduce spatial thinking, particularly if spatial thinking is considered a fundamental cognitive process underlying STEM success (Harris, Newcombe, & Hirsh-Pasek, 2013; Sekiyama, Kinoshita, & Soshi, 2014). Sekiyama et al. (2014) suggest that children aged around seven to eight years fall in a transition period for spatial thinking, wherein more mental processing emerges from the earlier physical and embodied approaches. Supporting this transition, Sekiyama et al. (2014) showed a decrease in hand gestures during mental rotation from the ages seven to eight years, contrasting with younger children, who all used hands to support mental rotation, and older children and adults, who rarely use hands. Thus, implementing embodied spatial training at this developmental stage should facilitate spatial thinking.

Several aspects of Think3d! provide embodied links to spatial thinking, both generally and in mathematics. For spatial thinking, generally, students physically transform the 2D paper into a 3D object and back to paper by both folding and unfolding. This embodies the classic Mental Paper Folding task (Shepard & Feng, 1972). As they construct the object, they rotate intermediate forms of it, similar to Mental Rotation (Shepard & Metzler, 1971) or Purdue Rotations tests (Guay, 1976). In other words, the actions result in internal-dynamic, 2D-to-3D transformations seen in STEM contexts (Newcombe & Shipley, 2015). For spatial thinking within mathematics, children fold paper into fractional parts (e.g. in half) of the whole paper, pairing actions with fraction concepts that are further reinforced in diagrams. Their folds make different geometric shapes. They translate a diagram’s spatial instructions into actions and vice versa. Pop-up paper engineering actions differ when an object is symmetrical versus asymmetrical. In other words, the activities provide direct experience with mathematics concepts (Ionescu & Vasc, 2014). Supporting this, research shows that when talking about origami folding, people use spatial terms applicable to math (Taylor & Tenbrink, 2013; Tenbrink & Taylor, 2015). These include, but are not limited to spatial nouns such as *angle*, *baselines*, *corner*, *crease*, *direction*, *end*, *edge*, *line*, *position*, *shape*, *side*, *symmetry*, *three dimensions*, and *way* and spatial verbs such as *bisect* and *intersect* and other spatial terms that include *center*, *close*, *diagonal*, *down*, *even*, *halfway*, *in half*, *into*, *open*, *opposed*, *outside*, *over*, *overlap*, *straight*, *symmetrical*, *three-dimensional*, and *vertical*. For elementary-aged children, these activities are completed in a real-world context, as origami and paper engineering easily comprise regularly occurring activities for this age group.

Further, Think3d! activities provide practice translating and producing visual representations, externalizing mathematics concepts (Pape & Tchoshanov, 2001). Diagrams can aid scientific reasoning (Bauer & Johnson-Laird, 1993). Below, we make predictions on how we expect Think3d! to impact mathematical reasoning.

A previous small implementation of Think3d! in Grade 4 classrooms examined its impact on spatial thinking (Taylor & Hutton, 2013). Students completed six weeks of Think3d! activities with accompanying spatial thinking assessments before, during, and after implementation. Spatial assessments included Mental Paper Folding (Shepard & Feng, 1972), Mental Rotation (Shepard & Metzler, 1971), and Make-A-Dice tests (Taylor & Hutton, 2013). Results showed spatial thinking gains in Think3d! classrooms relative to control, specifically for Paper Folding and Make-A-Dice. The present study extends this work by exploring the potential impact of Think3d! on both spatial thinking and aspects of mathematics that engage spatial thinking, as well as by evaluating its effectiveness with a wider age range of students.

Think3d!’s spatial thinking tasks suggest where students might show gains in mathematical reasoning. We predicted gains on problems involving visual representations, given the practice kids get interpreting and producing diagrams. Similarly, we predicted gains on problems providing a real-world context, given kids have practiced spatial thinking in the context of origami and paper engineering. Finally, we predicted gains when mathematical concepts have a strong spatial component or when possible spatial solution strategies exist. To specifically examine these predictions, we developed a categorization of math problems that identifies those problems most likely affected by spatial training.

### Categorization of math problems

Students with strong spatial skills tend to perform well in mathematics. However, not all math problems require spatial thinking. Given previous findings suggesting that spatial training may have a more targeted, rather than generalized, impact on STEM outcomes, we developed a math categorization that would allow us to identify the types of math problems most likely positively impacted by embodied spatial training. This, in turn, allowed us to make predictions about the math problems that should show performance gains after completing Think3d!. We started by sourcing Common Core State Standard mathematics problems (http://www.commoncoresheets.com/), which organize mathematical problems by underlying concepts. However, any mathematical concept can be taught and/or assessed using a wide variety of visualizations (e.g. graphs, tables, pictures), question formats (e.g. multiple-choice or open response), and types of information (e.g. story-based or abstract calculation). While this variety provides a wide range of problems for practicing mathematical skills, it is problematic for identifying problems potentially impacted by a training program; there are endless combinations.

**Fig. 2:**
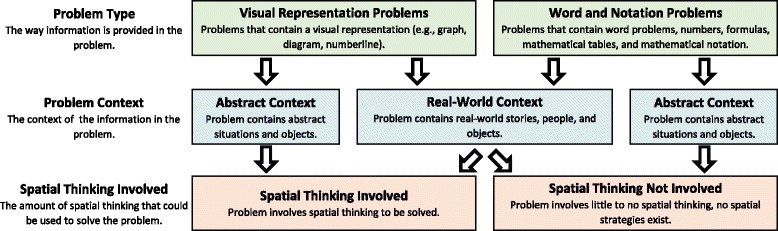
Mathematics categorization by problem type, problem context, and spatial thinking involvement. Example problems are provided in Fig. 3

Our math categorization included three categories (Fig. 2): problem type (*visual*, *word*/*notation*); problem context (*real-world*, *abstract*); and level of spatial thinking (*involved*, *not involved*). Problem type coded problems with visual representations, either visual (pictures, matrices, graphs, etc.) separately from those with verbal (word problems) or mathematical notation (fractions, mathematical tables, formulas, etc.). Problem context coded whether the problem dealt with real-world situations or lacked a real-world context (i.e. abstract). Level of spatial thinking coded whether spatial thinking could be involved in solving the problem. For example, problems involving graph interpretation were categorized as involving spatial thinking because students must understand that bar heights represent the *y*-axis values; using arrays or lattices to solve multiplication problems involves spatial thinking because students must use those 2D representations to calculate the product. While all math problems have at least some spatial properties (because we live in a 3D world), we categorized problems as not involving spatial thinking when there was no obvious spatial strategy or minor spatial properties involved in the problem. For example, problems in which students must use >, <, or = to compare numbers require an understanding of magnitude and have a directional component. However, these were categorized as not involving spatial thinking. Similarly, word problems that require two-digit multiplication might lead students to vertically stack the numbers, but these would be categorized as not involving spatial thinking. Using this categorization scheme, we developed brief assessments (ten items for Grades 3 and 4, eight items for Grades 5 and 6) that included problems representing all three factors. The problem type, problem context, and spatial thinking involved were not fully crossed (see Fig. 2), i.e. not all assessments contained every possible combination of factors. While a fully crossed design would have been ideal, the math assessment had to be feasible for elementary school students, i.e. not too many problems.

This categorization allowed us to make specific predictions about which mathematical problems should show performance gains after spatial training. Think3d! involves reading and creating visualizations, developing solutions in real-world contexts, and requires substantial spatial thinking. Therefore, performance gains should be found in math problems involving visual representations and real-world contexts and those incorporating spatial thinking.

### Current study

Students completed spatial and math assessments before and after participating in Think3d!. The assessments should help us determine how embodied spatial training impacts both spatial (Paper Folding and Purdue Rotations) and mathematical thinking, and combined spatial and mathematical thinking (Make-A-Dice). We predicted the following: (1) Accuracy will improve (from pre-test to post-test) for spatial tests (Paper Folding and Purdue Rotations) and combined spatial with mathematics test (Make-A-Dice). (2) For mathematics, improvements will be seen for targeted problem types. These would include visual representation problems, real-world context problems, and problems that use spatial thinking. (3) Accuracy on the spatial and combined spatial with mathematics tests will predict mathematics accuracy. It should be noted that there was no control group (i.e. all students participated in Think3d!). While an active control group is desirable, this implementation occurred during iterative development of both Think3d! and the assessments. As such, we needed to be sensitive to interruptions created by data collection within schools. Instead this research focused on pre-test and post-test differences for spatial thinking and targeted mathematics problems. These problems have the greatest utility for examining whether embodied spatial training impacts spatial thinking and mathematics outcomes. Mathematics problems that we would not expect embodied spatial training to impact were used for comparison and to rule out a simple practice effect explanation.

## Methods

### Participants

Ninety-two students in Grades 3 to 6 from two rural New England schools participated in the Think3d! program in 2014–2015. Of those students, 86 completed both the pre-tests and post-tests from at least one of the measures and were included in the analyses (Table 1).

**Table 1:** Number of students who completed the Think3d! program in 2014–2015

School	Grade 3		Grade 4		Grade 5		Grade 6	
	Girls	Boys	Girls	Boys	Girls	Boys	Girls	Boys
School A	6	6	3	8	9	6	6	6
School B	4	6	10	2	4	4	3	3
By gender	10	12	13	10	13	10	9	9
By grade	22		23		23		18	

### Materials

Students completed two sets of four pre-assessments and post-assessments: (1) a mathematics test; (2) a Make-A-Dice test; (3) a Paper Folding test; and, (4) a Purdue Rotations test. All grades completed the same Make-A-Dice, Paper Folding, and Purdue Rotations tests; however, each grade completed a grade-appropriate math assessment that used standards targeting one grade-level below the student’s current grade. Proportion correct (or accuracy), as a function of problems attempted, was calculated for all measures.

#### Mathematics test

**Fig. 3:**
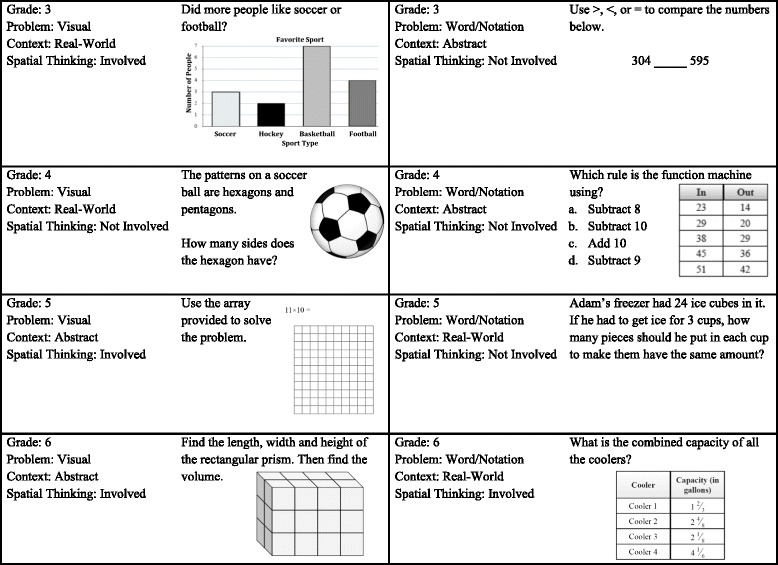
Example math problems for Grades 3–6, with their categorization based on problem type, context, and spatial thinking involved. Sourced from http://www.commoncoresheets.com

The math assessments consisted of eight to ten mathematics problems sourced from Common Core mathematics worksheets (see examples in Fig. 3). Since students were assessed at different times across the school year, grade-specific math tests used problems targeting standards from one grade younger (e.g. Grade 3 students completed Grade 2 Common Core standards). This ensured some familiarity with concepts being tested. Math problems varied on the problem type (visual representation items versus word/notation items), the problem context (real-world items versus abstract items), and level of spatial thinking (items involving spatial thinking versus those not involving spatial thinking). The number of items from each category differed between the grades but not between the pre-tests and post-tests within a grade (e.g. three abstract items on Grade 3 pre-tests and post-tests, but two abstract items on Grade 4 pre-tests and post-tests). Pre-test and post-test versions for a given grade matched problems to ensure similar difficulty level (e.g. two-digit multiplication was used as word/notation, abstract, non-spatial problem in both pre-tests and post-tests). Each test item was scored for whether the student attempted the problem and, if attempted, the solution accuracy. For Grade 3, Cronbach’s alpha was moderate (ten questions; pre-test *α* = 0.54; post-test *α* = 0.61) and test performance was correlated, *r*(21) = 0.63, *p* < 0.01. For Grade 4, Cronbach’s alpha was moderate to high (ten questions; pre-test *α* = 0.66; post-test *α* = 0.73) and test performance was correlated, *r*(23) = 0.45, *p* < 0.05. For Grade 5, Cronbach’s alpha was moderate (eight questions; pre-test *α* = 0.55; post-test *α* = 0.51) and test performance was correlated, *r*(21) = 0.69, *p* < 0.01. For Grade 6, Cronbach’s alpha was moderate (eight questions; pre-test *α* = 0.70; post-test *α* = 0.60) and test performance was correlated, *r*(18) = 0.73, *p* < 0.001.

#### Make-A-Dice test

**Fig. 4:**
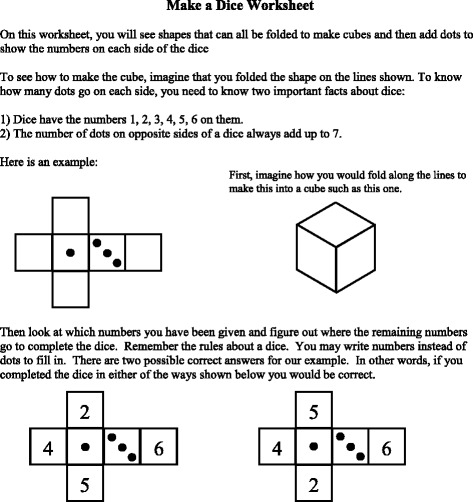
Make-A-Dice instructions and example problem with correct answer

The Make-A-Dice test was designed to test spatial visualization combined with math aptitude. Make-A-Dice items (Taylor & Hutton, 2013) present a flattened cube diagram (cube net) with two numbered squares/sides (Fig. 4). One imagines folding the cube net into a cube and then fills in numbers on the remaining squares/sides, following two rules: use numbers 1–6 only once and opposite sides of the cube must add to 7. Thus, for each item, four squares/sides need to be completed, leading to a maximum score of four points per item. For the current study, each test included eight items. Pre-test and post-test versions were matched for difficulty by using the same cube nets for five problems on each and by using different cube nets that were matched for number of folds required for the remaining three problems. Cronbach’s alpha was high for both versions (pre-test *α* = 0.73; post-test *α* = 0.86) and test performance was correlated, *r*(81) = 0.63, *p* < 0.001.

#### Short form Paper Folding test

The Paper Folding test assesses spatial visualization or the ability to imagine 3D objects (Ekstrom, French, Harman, & Dermen, 1976). Each item shows a series of diagrams depicting a piece of paper being folded one to three times and then a hole punched through it. Students choose the diagram that correctly shows what the hole-configuration would look like after the paper is unfolded. Here, students completed short versions of the Paper Folding test that included six items (pre-test: 1, 3, 5, 7, 9, 10; post-test: 2, 4, 6, 8, 20, 11). We selected these items in an attempt to match the pre-tests and post-tests for difficulty, matching for factors such as number of folds and presence of occlusion. Cronbach’s alpha was low (pre-test *α* = 0.45; post-test *α* = 0.37) and test performance was correlated, *r*(84) = 0.41, *p* < 0.001.

#### Purdue Rotations test

In the Purdue Rotations test (Guay, 1976), students solve a spatial analogy by viewing a diagram of a 3D object before and after rotation. Students then see another 3D object and select the option depicting that object rotated in the same manner as the first object. This test evaluates mental rotation skill and reasoning by analogy. Students completed short versions of the test with ten items. The pre-test included ten re-drawn items from the original test (items 1, 2, 5, 7, 8, 9, 11, 14, 16, 19) and the post-test used ten newly developed items (using novel 3D objects, but with rotations matching the original test). The original Purdue Rotations test systematically increases in difficulty and our item selection provided a range of difficulty levels. The post-test items were matched to the pre-test items for both number of rotations in the problem and number of rotations in each response item. Cronbach’s alpha was low (pre-test *α* = 0.40; post-test *α* = −0.05) and test performance was not significantly correlated, *r*(81) = 0.18, *p* < 0.10. While students attempted nearly all of the Purdue Rotations items, it was clear from their performance that the Purdue Rotation test was very difficult for this age range.

### Procedure

Students completed pre-assessments one week prior to the six weeks of Think3d! and finished with post-assessments one week after Think3d!. The pre-tests and post-tests consisted of different versions of four assessments, each with different time limits: (1) an eight-item to ten-item (8 min) mathematics test; (2) an eight-item Make-A-Dice test (5 min); (3) a six-item Paper Folding test (6 min); and, (4) a ten-item Purdue Rotations test (10 min).

#### Think3d! embodied spatial training program

Think3d! presents six sets of spatial thinking challenges incorporated into origami and pop-up paper engineering activities. Each set introduces a specific concept and includes multiple challenges designed to be part of an explicit instructional session (hereafter referred to as “explicit session”) and other challenges designed to be either explicit sessions or between-session challenges for students to undertake in free-choice time (hereafter referred to as “between challenges”). Although the specific activities vary for each set, all of the activities involve interpreting or producing diagrams, mapping diagram information onto real-world actions (e.g. fold, turn, or cut paper), carrying out actions, evaluating result of actions, and discussing diagrams or actions with peers. In other words, the lessons have kids combine visual perception and action in the service of understanding 2D to 3D transformations, fitting well in standard conceptions of embodied learning (Barsalou, 2008, 2010).

Prior to implementing Think3d!, all teachers received an in-person training session on program implementation. They received all materials, allowing them to preview the activities. Additionally, research staff answered teacher implementation questions by email or phone. As is frequently the case, research conducted in classrooms needed to fit within regularly scheduled classroom activities. To increase implementation feasibility within limited instructional time during school days, teachers had some flexibility in how they implemented the program. Individual teachers decided the specific number (minimum six, one for each set) and duration of explicit sessions to fit with other instructional demands. Explicit instruction sessions were designed to last approximately 45 min, but included materials to cover longer sessions and/or kids who work more quickly. All classrooms worked within the six-week implementation time frame. Teachers who chose to have more explicit sessions either extended the explicit session materials to cover more days or a longer time or they used the between session materials explicitly. Additionally, classrooms had between session materials accessible to students for gap times in classroom schedules (e.g. before school, student free-choice, rainy recess, etc.). In summary, all students participated in six explicit sessions and had between session materials accessible. It is likely that students had more Think3d! practice beyond the six explicit sessions, but given that the program encourages self-motivated practice individual student differences likely emerged and could not be tracked.

For explicit instruction sessions, students received all materials needed for the spatial challenges. If the challenge involved using labeled diagrams to make paper objects, they received diagrammed instructions, enough paper to allow multiple attempts for each object, and scissors, if relevant. If the challenge involved reverse engineering, i.e. students figured out the folding and/or cutting sequence to recreate a paper object, they received complete objects that could be deconstructed, paper and/or scissors to iteratively test theories on how to construct the object, and then materials to diagram how to make the object. Students worked in groups for all sessions and were encouraged to discuss problems and solutions with one another. Between sessions involved additional challenges, promoting additional practice. These were similar in nature to the explicit sessions. Throughout each explicit session, the teacher circulated to provide tips for students stuck on a task. Teachers were instructed to provide guidance by pointing out correspondences between instructions/available models and the materials the students were working with rather than explicitly telling students how to proceed.

**Fig. 5:**
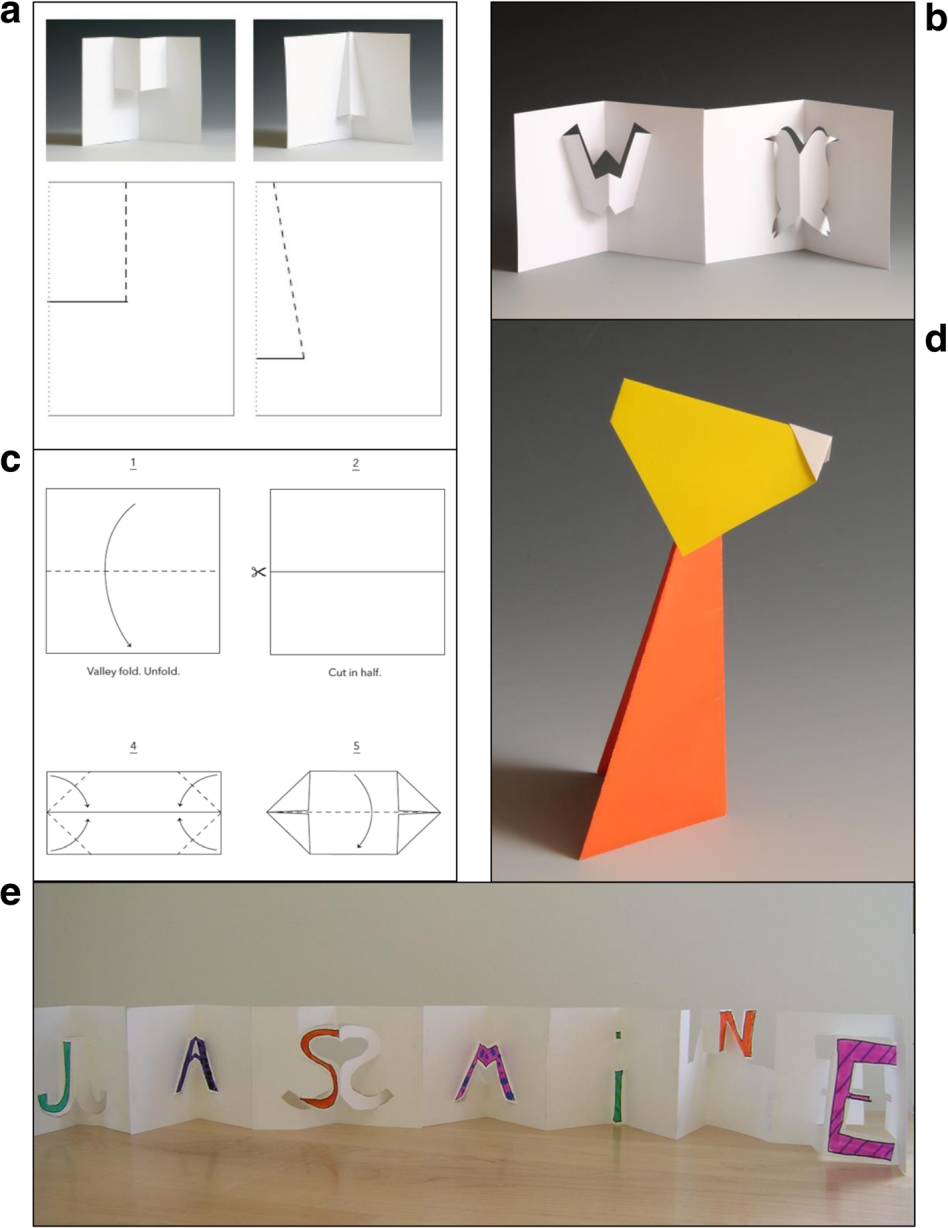
Elements of the Think3d! program. **a** Portion of single sheet paper engineering instructions leading students through outcomes when varying folds and cuts. **b** Student-created pop-up after completing lessons illustrated in a). **c** Portion of origami instructions providing visual folding language. **d** Origami nodding dog for reverse engineering. **e** Student-created name, extending reverse engineering of small set of provided pop-up letters

The first two explicit sessions and the first two between challenges involved origami. The first of each involved interpreting visual instructions and learning origami symbols (e.g. valley or mountain fold) to make simple origami objects (see Fig. 5c). Students worked in small groups to figure out the diagrams and freely provided help to others. The second explicit and between sessions involved exploration and experimentation with knowledge gained in the earlier lessons. This involved reverse engineering, figuring out how to fold an object by exploring completed objects, and/or exploratory modifications of known origami objects (see Fig. 5d).

The next explicit and between sessions involved single-sheet paper engineering. Again, the early sessions involved interpreting diagrams (see Fig. 5a). Diagram instructions varied the angle of the fold, the shape of the cut, and the number of cuts. Through folding and cutting, students saw how these variations affected their 3D pop-ups. Over time and in line with the instruction format (diagrams juxtaposed with resultant pop-ups), students practiced mental paper folding, making the connection between 2D diagram and 3D form (see Fig. 5a). Unplanned exploration naturally shifted to intentional design as students’ ability to visualize cause and effect in pop-up engineering grew (see Fig. 5b). The later lessons, paralleling the origami lessons, again had students explore and experiment. Students received pop-up models – a set of alphabet letters comprising three vowels and three consonants. They explored these models to make their own letters and words, extending from the sub-set provided to create their own name or other words (see Fig. 5e). These lessons focused on intentional exploration, but built on earlier paper-engineering lessons. In this iterative process, students could analyze cause and effect: diagramming, realizing the resultant pop-up, and modifying the diagram based on the gap between actual and intended outcome. This intentional exploration and design practices took them from physical folding to mental folding and visualization.

## Results

Although this study did not include a control group, targeted differences between the pre-tests and post-tests should provide a first step towards evaluating the consequences of the embodied spatial training on spatial and mathematics outcomes. Specifically, we predicted targeted improvements on math problems with visual representations, real-world contexts, and required/involved spatial thinking. Pre-test and post-test performance on other problem types served as a comparison.

### Mathematics test

Eighty-three students from two schools completed math pre-tests and post-tests (Grade 3 = 21, Grade 4 = 23; Grade 5 = 21; Grade 6 = 18; girls = 43; boys = 40). Changes across the tests (pre; post) and the interaction between test and grades (3, 4, 5, 6) were investigated with linear mixed-effects models, using the “nlme” package in R version 3.1.2 (Pinheiro, Bates, DebRoy, Sarkar, & R Core Team, 2014). The dependent variable was percent accuracy of problems attempted. While we included grade as a between-subjects factor in the models, each grade completed grade-specific math tests. As such, grade main effects would not be interpretable and will not be discussed. We report interactions between test and grade as indicators of developmental (operationalized as grade) differences in Think3d! participation.

**Fig. 6:**
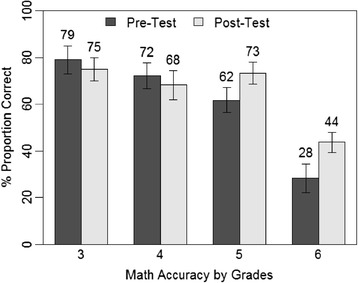
Proportion correct by grade for the math test, with mean values for each test and standard error bars

Students attempted a high proportion of math problems in both the pre-test (*M* = 88%) and post-test (*M* = 91%; Table 2). Overall math accuracy marginally increased from the pre- (*M* = 62%) to post-test (*M* = 66%), *F*(1, 79) = 3.87, *p* = 0.05, *η* ^*2*^ = 0.01. Test type and grade interacted, *F*(3, 79) = 5.85, *p* < 0.01, *η* ^*2*^ = 0.05. The upper two grades improved in accuracy, while Grade 3 and Grade 4 accuracy decreased slightly (Fig. 6).

**Table 2:** Percent of attempted items for each test, grade, and measure

	Mathematics test		Make-A-Dice test		Paper Folding test		Purdue Rotations test	
Grade	Pre	Post	Pre	Post	Pre	Post	Pre	Post
3	96%	90%	58%	65%	94%	98%	97%	90%
4	93%	97%	72%	86%	91%	99%	92%	97%
5	95%	97%	68%	74%	98%	100%	98%	98%
6	65%	76%	63%	84%	94%	100%	91%	92%
Mean	88%	91%	66%	78%	94%	99%	94%	94%

**Fig. 7:**
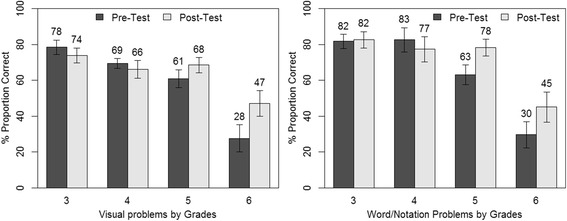
Proportion correct for visual representation problems (*left*) and word/notation representation problems (*right*) by grade for the math test, with mean values for each test and standard error bars

Although overall accuracy is important, not all math problems involve spatial thinking; we predicted math performance differences based on our math categorization. Specifically, we expected embodied spatial training to positively impact visual problems, but not necessarily word/notation problems. While, accuracy was constant across the pre-tests (*M* = 61%) and post-tests (*M* = 65%) for visual problems, an interaction between test and grade qualified this effect, *F*(3, 77) = 3.38, *p* < 0.05, *η* ^*2*^ = 0.04. The upper two grades improved on visual problems, while the lower two did not. For word/notation problems, accuracy was constant across the pre-tests (*M* = 66%) and post-tests (*M* = 72%), and the interaction between test and grade was not significant (Fig. 7).

**Fig. 8:**
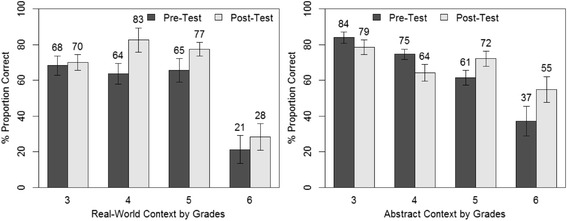
Proportion correct for real-world context problems (*left*) and abstract context problems (*right*) by grade for the math test, with mean values for each test and standard error bars

We also expected improvements for real-world, but not for abstract context problems. Results support this prediction, although older students also improved on abstract problems. Accuracy improved on real-world problems (pre-test *M* = 56%, post-test *M* = 67%), *F*(1, 77) = 8.67, *p* < 0.01, *η* ^*2*^ = 0.03. There was no interaction between test and grade. In contrast, accuracy remained constant for abstract context problems (pre-test *M* = 65%, post-test *M* = 68%). Test interacted with grade for abstract problem accuracy, *F*(3, 79) = 5.11, *p* < 0.01, *η* ^*2*^ = 0.06, as lower grade accuracy decreased while upper grades showed improved accuracy (Fig. 8).

**Fig. 9:**
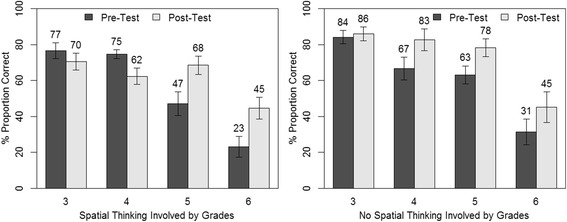
Proportion correct for problems involving spatial thinking (*left*) and non-spatial problems (*right*) by grade for the math test, with mean values for each test and standard error bars

We expected improvements on problems that involved spatial thinking, but problems not involving spatial thinking would not improve. Problems involving spatial thinking did not show overall improvement (pre-test *M* = 57%, post-test *M* = 62%), but test interacted with grade, *F*(3, 79) = 10.35, *p* < 0.001, *η* ^*2*^ = 0.11, showing that the upper two grades improved greatly on spatial thinking problems. For problems not involving spatial thinking, accuracy significantly improved across the tests (pre-test *M* = 63%, post-test *M* = 74%), *F*(1, 77) = 12.39, *p* < 0.001, *η* ^*2*^ = 0.05, but there was no interaction between test and grade (Fig. 9).

### Spatial thinking assessments

Analyses for the spatial thinking assessments matched those used for the math assessments. Only students who completed both pre-tests and post-tests for an assessment are included in the analyses, leaving different numbers of students for each assessment. Because all students completed the same spatial assessments, we discuss differences based on grade.

#### Paper Folding test

**Fig. 10:**
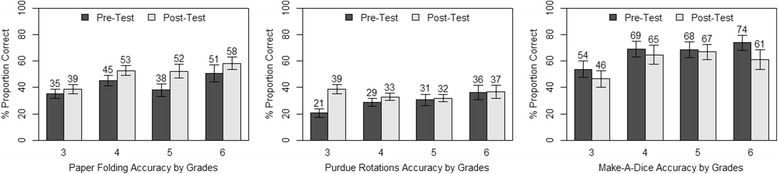
Proportion correct by grade for Paper Folding (*left*), Purdue Rotations (*center*), and Make-A-Dice (*right*), with mean values for each test and standard error bars

Eighty-four students completed the pre- and post- Paper Folding tests (Grade 3 = 21, Grade 4 = 23; Grade 5 = 21; Grade 6 = 18; girls = 43; boys = 40). Students attempted a high proportion of Paper Folding problems in both the pre-test (*M* = 94%) and post-test (*M* = 99%; Table 2). Paper Folding accuracy increased across tests (pre-test *M* = 42%, post-test *M* = 50%), *F*(1, 79) = 11.05, *p* < 0.01, *η* ^*2*^ = 0.04. Accuracy increased across grades, *F*(3, 79) = 3.62, *p* < 0.05, *η* ^*2*^ = 0.09. Grade 3 students’ accuracy (*M* = 37%) was significantly lower than Grades 4 (*M* = 49%) and 6 (*M* = 54%). Grade 5 students’ accuracy (*M* = 45%) did not differ from the other grades. Test type did not interact with grade (Fig. 10).

#### Purdue Rotations test

Eighty students completed the pre- and post- Purdue Rotations tests (Grade 3 = 20, Grade 4 = 23; Grade 5 = 19; Grade 6 = 18; girls = 42; boys = 38). Students attempted a high proportion of Purdue Rotations problems (pre-test *M* = 94%, post-test *M* = 94%; Table 2). Purdue Rotations accuracy increased from pre-test (*M* = 29%) to post-test (*M* = 35%), *F*(1, 76) = 7.21, *p* < 0.01, *η* ^*2*^ = 0.03, but overall remained quite low. Accuracy slightly, but non-significantly, increased across grades, (*M*s = 30% Grade 3, 31% Grade 4, 31% Grade 5, 36% Grade 6). Test type interacted with grade, *F*(3, 76) = 3.27, *p* < 0.05, *η* ^*2*^ = 0.04, as grade 3 student performance greatly increased across tests (Fig. 10).

### Combined spatial thinking and math assessments: Make-A-Dice test

Seventy-nine students completed pre- and post- Make-A-Dice tests (Grade 3 = 19, Grade 4 = 23; Grade 5 = 19; Grade 6 = 18; girls = 41; boys = 38). Attempt rates were high (pre-test *M* = 66%, post-test *M* = 78%; Table 2). Make-A-Dice accuracy decreased across tests (pre-test *M* = 66%, post-test *M* = 60%), *F*(1, 75) = 6.54, *p* < 0.05, *η* ^*2*^ = 0.06. Third-graders (*M* = 50%) had lower accuracy compared to the other grades (*M*s = 67% grade 4, 68% grade 5, 68% grade 6), but accuracy did not significantly differ by grade. Test did not interact with grade (Fig. 10).

### Predicting math accuracy

In order to assess whether changes in spatial thinking predicted changes in mathematics reasoning, we used multiple linear regressions to predict change in math accuracy. Predictors included grade, and pre-post change in spatial task (Make-a-Dice, Paper Folding, Purdue Rotations) accuracy, using the “stats” package in R version 3.1.2. It should be noted that change in accuracy on the spatial tests are not correlated: change in Make-A-Dice is not correlated with change in Paper Folding, *r*(77) = 0.02, *p* = 0.86, change in Make-A-Dice is not correlated with change in Purdue Rotations, *r*(75) = −0.04, *p* = 0.72, and change in Paper Folding is not correlated with change in Purdue Rotation, *r*(78) = 0.00, *p* = 1.00.

**Fig. 11:**
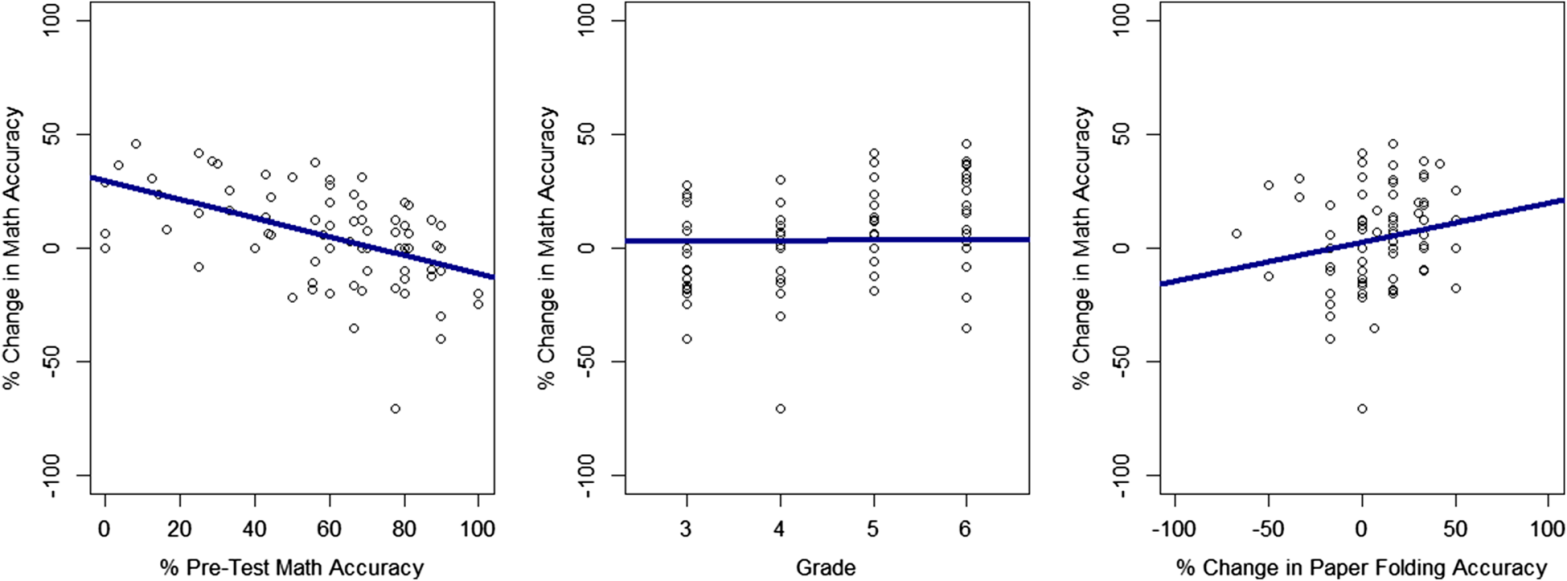
Percent change in math accuracy by pre-test math accuracy (*left*), grades (*center*), and change in Paper Folding accuracy (*right*), with regression lines

An initial regression model used pre-test math accuracy (fixed effect) and grades (mixed effect) to predict math accuracy change (accuracy for all measures is proportion correct). Students’ predicted *change* in math accuracy was equal to 23.62 (intercept) − 0.39 (pre-test math accuracy) − 1.06 (grade). This model reveals that the average change in math accuracy is 23.6% (when pre-test math accuracy is zero), for every 0.39% increase in pre-test math accuracy the model predicts a 1% decrease in change in math accuracy (if grade is held constant), and for every increase in grade the model predicts a 1.06% decrease in math accuracy change (if pre-test math accuracy is held constant). As pre-test math accuracy increases the change in math accuracy decreases, as there is less opportunity for gains, and as grade increases changes in math accuracy also decreases. However, only pre-test accuracy (*t* = −3.69, *p* < 0.001) was a significant predictor. Figure 11 illustrates this relationship more clearly. The other regression models presented should be similarly interpreted.

Another regression model used change in accuracy for the three spatial tests (fixed effects) and pre-test math accuracy (fixed effect) to predict change in math accuracy. Students’ predicted *change* in math accuracy was equal to 29.02 − 0.44 (pre-test math accuracy) + 3.10 (gender = male) + 0.06 (change in Make-A-Dice accuracy) + 0.20 (change in Paper Folding accuracy) − 0.02 (change in Purdue Rotations accuracy). Change in Paper Folding accuracy (*t* = 2.19, *p* < 0.05) and pre-test math accuracy (*t* = −4.83, *p* < 0.001) were both significant predictors. In a regression model with only pre-test math accuracy and change in Paper Folding accuracy, students’ predicted *change* in math accuracy was equal to 29.07 − 0.43 (pre-test math accuracy) + 0.20 (change in Paper Folding accuracy). Change in Paper Folding accuracy (*t* = 2.43, *p* < 0.05) and pre-test math accuracy (*t* = −5.26, *p* < 0.001) were both significant predictors. This model indicates that as pre-test math accuracy increases the change in math accuracy decreases, as there is less opportunity for gains, but as change in Paper Folding accuracy increases changes in math accuracy increases (Fig. 11).

**Fig. 12:**
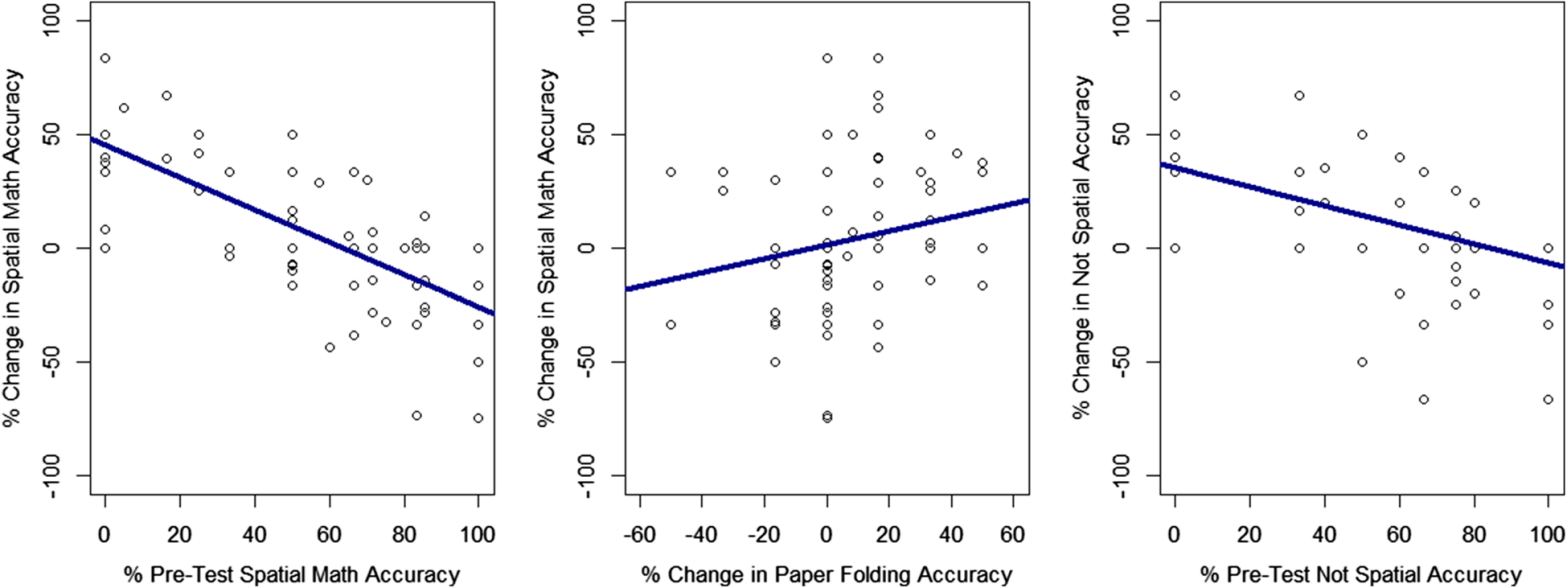
Percent change in math accuracy, only for problems that involve spatial thinking, by pre-test math accuracy, only for problems that involve spatial thinking (*left*), and change in Paper Folding accuracy (*center*), with regression lines. Percent change in math accuracy, only for problems that do not involve spatial thinking, by pre-test math accuracy, only for problems that do not involve spatial thinking (*right*), with regression lines

A regression model was created using change in accuracy for the three spatial tests (fixed effects) to predict change in spatial math accuracy (items that involved spatial thinking). Students’ predicted *change* in spatial math accuracy was equal to 39.82 − 0.66 (pre-test spatial math accuracy) + 0.12 (change in Make-A-Dice accuracy) + 0.25 (change in Paper Folding accuracy) + 0.13 (change in Purdue Rotations accuracy). Pre-test spatial math accuracy (*t* = −7.82, *p* < 0.001) and change in Paper Folding accuracy (*t* = 2.30, *p* < 0.05) were both significant predictors. This model indicates that as pre-test spatial math accuracy increases the change in spatial math accuracy decreases, but as change in Paper Folding accuracy increases change in spatial math accuracy increases (Fig. 12).

Another regression model used change in accuracy for the three spatial tests (fixed effects) to predict change in non-spatial math accuracy (items that did not involve spatial thinking). Students’ predicted *change* in non-spatial math accuracy was equal to 35.92 − 0.45 (pre-test non-spatial math accuracy) − 0.04 (change in Make-A-Dice accuracy) + 0.19 (change in Paper Folding accuracy) − 0.16 (change in Purdue Rotations accuracy). Only pre-test score on non-spatial math problems (*t* = −4.21, *p* < 0.001) was a significant predictor. This model indicates that as pre-test non-spatial math accuracy increases the change in non-spatial math accuracy decreases (Fig. 12).

In sum, these models reveal that overall math improvements are predicted by improvements on the Paper Folding test, a near transfer test of the origami and paper engineering diagram interpretation skills developed by Think3d!. More specifically, improvements on the Paper Folding test are predictive of improvements on math problems that involve spatial thinking over those that do not. As predicted, change in performance on one of the spatial measures significantly predicted change in math performance.

### Gender effects

In order to test whether there were any gender effects in the measures, between-subjects t-tests were run for each test (pre and post) for each measure. Accuracy did not significantly differ between genders in the pre-tests or post-tests for any of the measures (see Table 3 for mean accuracy rates).

**Table 3:** Percent accuracy for each test and measure by gender

	Mathematics test		Make-A-Dice test		Paper Folding test		Purdue Rotations test	
	Pre	Post	Pre	Post	Pre	Post	Pre	Post
Girls	63%	64%	69%	62%	44%	50%	27%	32%
Boys	60%	67%	64%	58%	40%	51%	31%	38%

## Discussion

The present work examined how an embodied spatial training program, Think3d!, impacted elementary students’ spatial and mathematical thinking. Students completed assessments evaluating spatial thinking (Paper Folding, Purdue Rotations), mathematical thinking, and the ability to combine spatial and mathematical thinking (Make-A-Dice), before and after participating in Think3d!. Since a control group was not feasible, we made targeted predictions about how spatial training would impact assessment accuracy. We predicted: (1) accuracy would improve (from pre-test to post-test) for spatial tests (Paper Folding, Purdue Rotations) and the task combining spatial thinking and math (Make-A-Dice); (2) within the mathematics test, accuracy on visual representation problems, real-world context problems, and problems that involve spatial thinking would increase; and (3) accuracy on the spatial tests and the combined spatial and math test would predict mathematics accuracy. These predictions along with their results will be discussed in turn. Although we did not have specific developmental predictions, we also explored developmental trends in the impact of embodied spatial training, operationalized by student grade.

### Embodied spatial training’s impact on spatial thinking

Both spatial tests (Paper Folding, Purdue Rotations) showed improvements with Think3d! participation, indicating that the program likely improves elementary students’ spatial thinking with visualizations. The Paper Folding and Purdue Rotations tests were used as near transfer tasks, as they involved visualizations similar to Think3d!’s origami visualizations and both tests showed improvements. Further, Purdue Rotations showed improvements despite being quite difficult for students, as evidenced by the overall low accuracy rates. While the present study cannot disentangle spatial task gains related to spatial training from those expected with practice, they are consistent with spatial thinking gains accompanying spatial training (Uttal, Meadow, et al., 2013). In summary, Paper Folding and Purdue Rotations performance likely improved after embodied spatial training, supporting evidence suggesting malleability of spatial thinking through training (Uttal, Meadow, et al., 2013; Uttal, Miller, et al., 2013).

### Embodied spatial training’s impact on task combining spatial thinking and mathematics

Contrary to our expectations and a previous implementation (Taylor & Hutton, 2013), the Make-A-Dice test did not show improvements. Make-A-Dice taxes working memory to a greater extent than Paper Folding and Purdue Rotations Tests as, in addition to imagining folding the cube, students must determine and remember which sides are opposite one another, assign numbers summing to 7, and keep in mind which numbers have already been used. Unlike the previous implementation carried out by the researchers (Taylor & Hutton, 2013), teachers carried out assessments in the present work. This may have lessened opportunities to clarify the Make-A-Dice instructions, which given the task complexity, could be problematic. Additionally, many younger students took time to draw small dots instead of writing numbers on each cube side, reducing the number of problems they could attempt, and/or making it more difficult to track which numbers had already been used.

### Embodied spatial training’s impact on mathematics

The present work explored the relationship between embodied spatial training and math outcomes in two ways. First, we examined math accuracy gains, comparing targeted problems expected to show change with embodied spatial training to those for which change would not be expected. Second, we used regression models to examine predictors, with a keen interest in spatial and combined spatial with math predictors of math performance.

#### Targeted math problems

Not all math problems involve spatial thinking, but those that do should, to a greater extent, be impacted by embodied spatial training. First, we examined performance on targeted math problem types, using our math categorization that identified problems by type (defined by its representational nature), context (real-world versus abstract), and whether the solution involved spatial thinking. Problems targeted for embodied spatial training improvement included visual representation problems, real-world context problems, and problems involving spatial thinking. Other studies have shown improvements on specific problem types. For example, Cheng and Mix (2014) found improvements for only missing term problems after mental rotation training.

Performance only partially supported these predictions. We first examined changes based on a problem’s representational format. As predicted, students’ math accuracy improved for problems including visual representations, but primarily for older kids (Grades 5 and 6). In contrast, word/notation problems showed no gains and did not interact with grade. Next, we examined problem context. Overall math performance improved on real-world, but not on abstract problems. Abstract, but not real-world, problem performance interacted with grade. Younger kids’ performance declined while older kids’ performance improved on abstract problems. Finally, we looked at problems that do or do not involve spatial thinking. For problems involving spatial thinking, older grades improved, but the younger grades did not. Problems not involving spatial thinking showed overall gains, but did not interact with grade. In sum, we used our math categorization to identify problems predicted to be impacted by embodied spatial training. Kids in older grades improved on problems involving visual representations and on those involving spatial thinking. Across all grades, students improved on real-world context problems.

Problems not expected to be affected by embodied spatial training served as our analytic comparison. These included the word/notation problem type, abstract context problems, and problems not requiring spatial thinking. Students showed some improvements on these problem types, as older grades improved on abstract context problems and students improved overall on problems not involving spatial thinking.

The feasibility of conducting research in classroom settings requires not placing too high an assessment burden on students, thereby protecting precious school day instructional time. As such, we could not include multiple versions of problems reflecting all possible combinations of our math categorization. The combined features of a specific math problem may better predict the impact of spatial training than individually identified features. For example, in the Grade 5 math test, students were asked to complete the following pattern: 56, 51, 46, 41, 36, __, __, using the following answers: a) 31, 26 b) 26, 21, c) 31, 36, and d) 33, 25, and the correct answer is a. This problem was classified as word/notation problem type, with abstract context, and non-spatial thinking involved. This type of problem, while classified as an abstract problem type can be solved spatially. Future work should parametrically explore how different factors involved in math problems impact the availability of spatial strategies. Our current implementation of Think3d! will further examine targeted math improvements both relative to other problem types and relative to control classrooms who have not had embodied spatial training. Overall our findings suggest that impact of spatial training on STEM outcomes should be considered alongside the potential for using spatial thinking within particular STEM domains and problem spaces.

The math results may suggest a developmental time point for which embodied spatial training could be more effective. Math improvements were more evident with older grades, although there is a possibility that these improvements were practice effects. If these improvements in older grades are replicable, then they could suggest that the impact of embodied spatial training on mathematics may either accrue over the course of development, emerge more dramatically at those points, or both. If so, this would be consistent with Sekiyama et al.’s (2014) results, suggesting a general transition from embodied to visual spatial thinking begins around seven to eight years of age with mastery at a later age.

#### Predicting math performance

We used regression models to explore whether spatial test performance would predict changes in math accuracy. These models also included grade to further explore a potential developmental time course for spatial training. Note that each grade had grade-appropriate math tests, so grade as a predictor was not a given. We predicted performance on a broader range of our spatial tests would predict math performance. This prediction was only partially born out; Paper Folding performance predicted change in math performance and change in math problems involving spatial thinking. Students who demonstrated the most improvement in Paper Folding also demonstrated the most improvement in mathematics. The Paper Folding test is the most similar to the spatial challenges within Think3d!. As such, these findings add to the literature showing spatial thinking’s relation to math outcomes (e.g., Lubinski & Benbow, 2006; Shea et al., 2001; Uttal, Miller, et al., 2013; Wai et al., 2009). In addition, these findings extend the literature to spatial training (e.g. Cheng & Mix, 2014).

In sum, our approaches of relating spatial training to math outcomes provide some positive indications for embodied spatial training. Improvements on the spatial and math assessments potentially derive from practice students had with spatial visualizations (both reading and creating diagrams) in Think3d!. This contention is supported by results showing that mental paper folding, a skill most practiced through the Think3d! activities, predicted math performance changes. While still tentative, our data hint that spatial thinking should be considered a basic cognitive skill and practiced in elementary school. Such training may have downstream positive effects relevant to understanding STEM concepts, particularly those concepts for which spatial thinking provides a route to understanding. Further, our data might suggest developmental differences within the third-grade to sixth-grade range relative to spatial training’s impact on math reasoning, as math accuracy gains were more evident in older grades. Additional research examining developmental differences across targeted changes in math performance with spatial training is needed.

While these findings show preliminary promise, they also suggest that embodied spatial training is not a panacea for training mathematical reasoning. At least for a six-week training program, the type of spatial thinking and the specific activities in the training program likely determine the extent to which that training impacts mathematical thinking. Teachers and parents should strive to encourage elementary-aged children to participate in a range of spatial thinking activities and/or training programs on a long-term basis, in order to see improvements in mathematics and, potentially, other STEM disciplines.

### Limitations and future work

The lack of a control group limits conclusions from this work. Not just a control group, but an active control group would have been ideal. With Think3d! activities and assessments now fully developed, our current implementation of Think3d! includes active control classrooms. Instead for the current phase of this work we took a targeted approach, in which the specific predictions for how Think3d! should and should not impact math performance provided a targeted comparison. Particularly for spatial assessments, it was difficult to disentangle training from practice effects.

Within our math categorization, it was not possible to develop math problems of similar levels of difficulty across all categorization levels. Given that these assessments were administered by teachers in classroom settings, the number of questions given to students within each assessment and the total time spent completing assessments had to fit within a typical class period. This resulted in mathematics assessments that limited the number of problems (with none for some grades) within each possible combination of the categorization. These challenges likely impacted our results and their interpretability. Future work should focus on specific math categories of interest or find methods of testing a larger number of math questions without fatiguing students. Follow-up studies will use redesigned mathematics tests that fully cross the categorization levels, although still limit total problem numbers to lessen assessment burden.

While we did find improvements in the Purdue Rotations after Think3d! participation, elementary students found these tests very difficult. A previous training study focusing on 3D spatial skills in Grades 8–11, found similar performance improvements on a simplified version of the Purdue Rotations test that also contained only ten items (Sorby, Drummer, & Molzon, 2006). Given that middle and high school students performed well but elementary students struggled on a simplified version of Purdue Rotations, future studies with elementary students should sample questions from the easiest Purdue Rotations questions and reduce the number of response items to select from (i.e. three items versus the standard five items).

Finally, we predicted improvements in the Make-A-Dice test, but did not find improvements. This could be due to either the heavy cognitive demands or to instructional misunderstandings, points that cannot be disentangled in the current data. Follow-up studies will feature simplified instructions, hopefully increasing the fidelity of implementation of this measure within classrooms, and redesigned items for the Make-A-Dice test, and redesigned mathematics tests, to address these issues.

## Conclusions

This study is the first to examine effects of embodied spatial training on elementary students’ math performance within a classroom setting. While previous work has linked spatial thinking and mathematics through longitudinal data (e.g. Lubinski & Benbow, 2006; Shea et al., 2001; Wai et al., 2009) and demonstrated how pencil-and-paper mental rotation training can improve young children’s math performance (Cheng & Mix, 2014), this is the first work to explicitly apply embodied training. Results showed some spatial thinking gains, adding to the growing body of literature that improving spatial thinking is possible through training (Uttal, Meadow, et al., 2013; Uttal, Miller, et al., 2013). Results also showed promising gains on real-world, visual, and spatial thinking math problems, further supporting the importance of spatial thinking skills for learning in multiple STEM fields. So, despite spatial training being missing from the current education system (National Research Council, 2006), embodied spatial training may provide a means to train a fundamental cognitive skill, spatial thinking, which in turn has important implications for mathematics learning. Fostering spatial thinking and mathematics learning in elementary school could contribute to a downstream ripple effect, improving students’ interest and success in STEM subjects throughout their education and into their careers.

## Abbreviations

2D: Two-dimensional
3D: Three-dimensional
STEM: Science, Technology, Engineering, and Mathematics
